# Supplementary material for: Relationship among state reopening policies, health outcomes and economic recovery through first wave of the COVID-19 pandemic in the U.S
Source: PLoS One. 2021 Nov 18;16(11):e0260015. doi: 10.1371/journal.pone.0260015 (PMC8601438; doi:10.1371/journal.pone.0260015)
Supplement: S1 File — (PDF) [file pone.0260015.s001.pdf]

Relationship among state reopening policies, health outcomes and economic recovery through first wave of the COVID-19 pandemic in the U.S.

Supporting Information

S1. Limitations

The limitations of this analysis must be considered when our results are interpreted. One limitation is our classification of state lockdown and reopening interventions into categories based on the CDC/White House guidelines. State decisions do not always match the guidelines perfectly. One issue is that each phase of the CDC/White House guidelines includes a number of different requirements, and for some states it is not obvious to determine the exact date when all requirements for a phase transition were satisfied. Moreover, in Pennsylvania and several other states interventions were not implemented in all counties at the same time and were often conditional on the state of COVID-19 spread or other factors. Another limitation is the retrospective and observational nature of data in this and other COVID-19 studies, which makes it difficult to identify the effect of unobserved factors [1]. In particular, the variation in consumer card spending may not be related with government mandates alone but affected also by other state-specific factors such as unemployment levels, population densities, local incidence and

prevalence of COVID-19, changing hospital capacities, or even an over-emphasis on attempting to return to a sense of normality. Although we have added state political majority and state median income to our difference-in-differences estimate to account for state heterogeneity, we acknowledge that other unobserved confounding factors may be present.

S2. Results

Trends on COVID-19 spread and consumer card spending for all 50 states

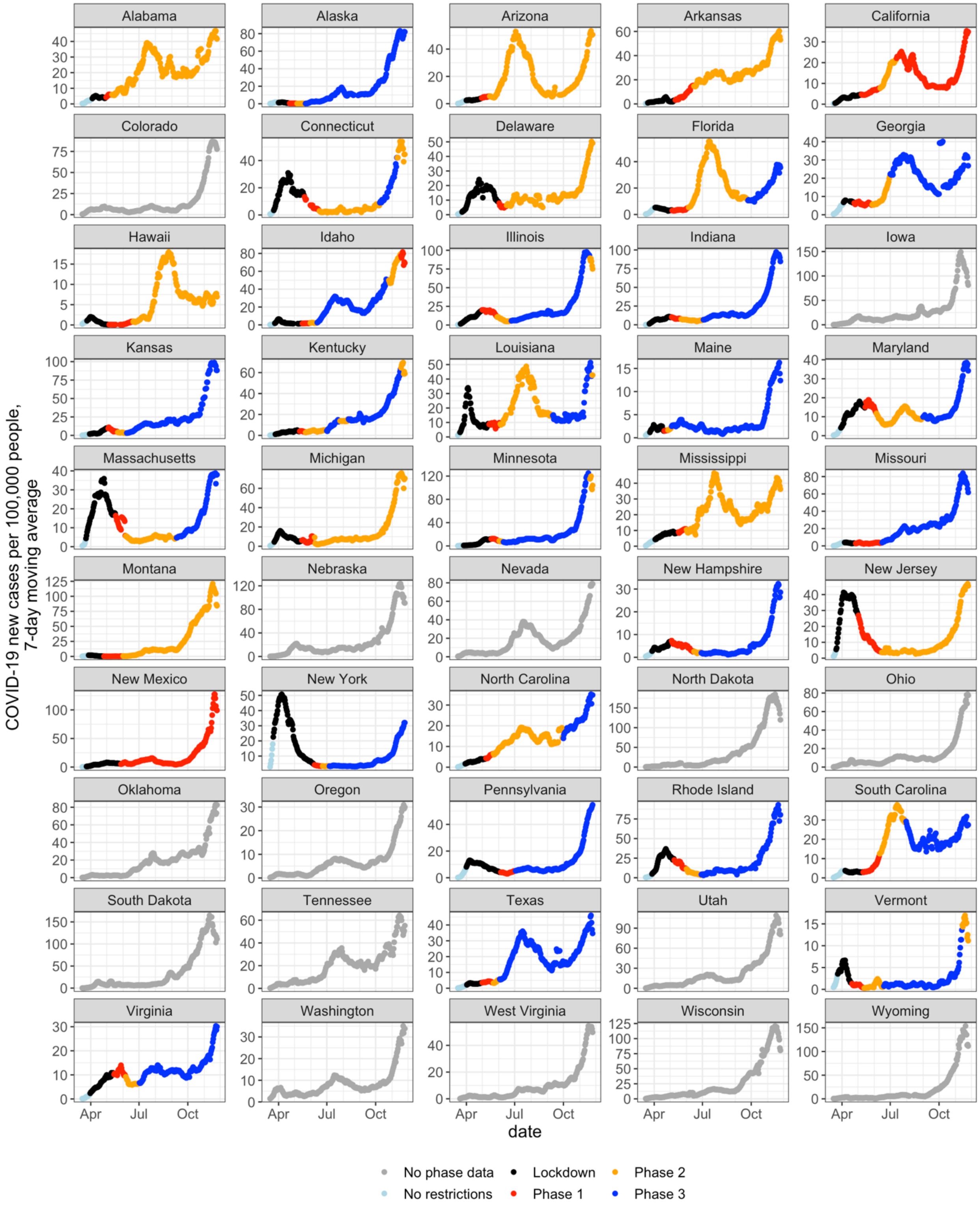

Figure S1. Seven-day moving average of new daily COVID-19 cases per 100,000 people per state. The colors represent our classification of state interventions into the three phases of the White House/CDC reopening guidelines (where phase classification could be performed).

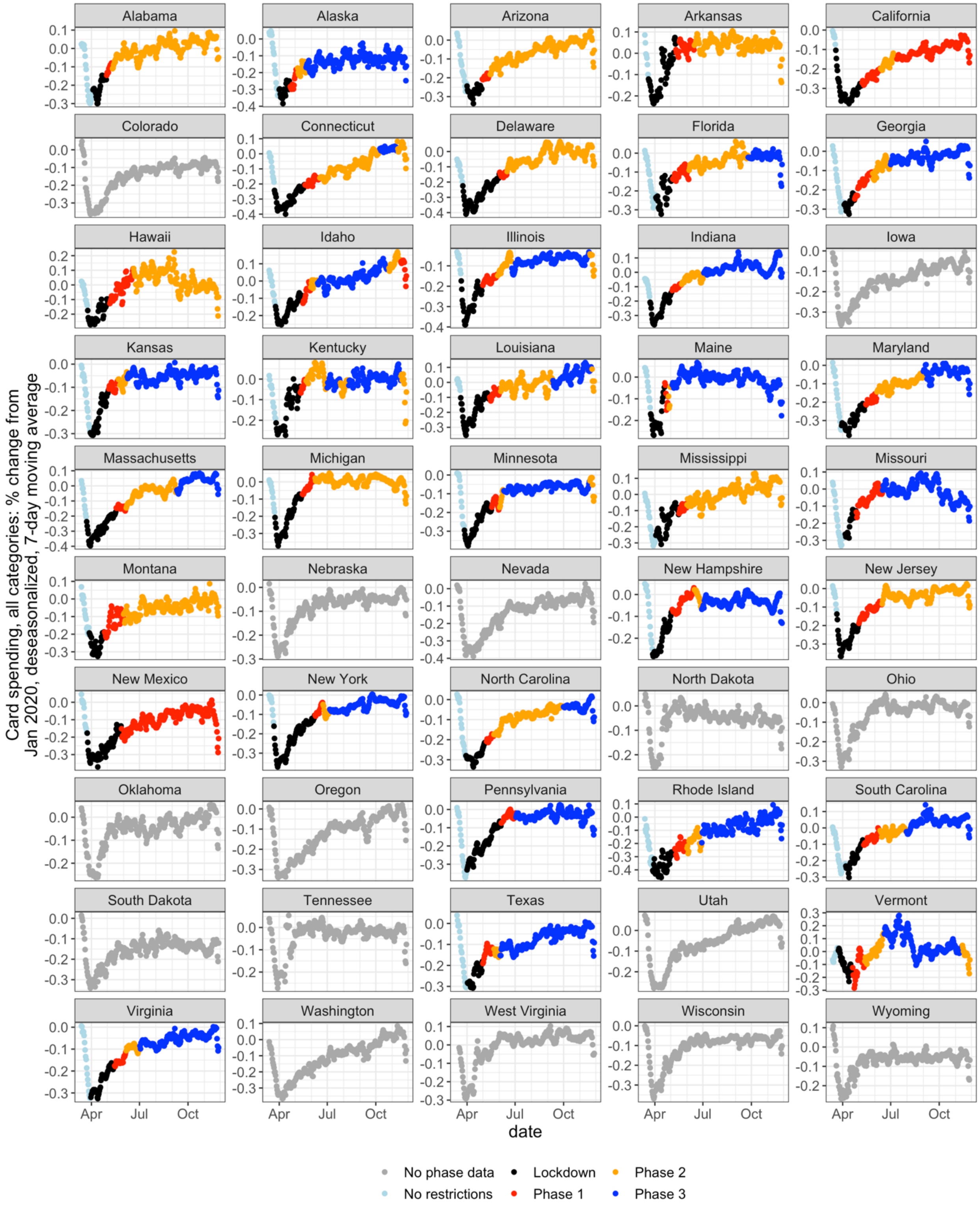

Figure S2. Seven-day moving average of de-seasonalized change in consumer card spending (relative to January 2020) over time. The colors represent our classification of state interventions where phase classification could be performed.

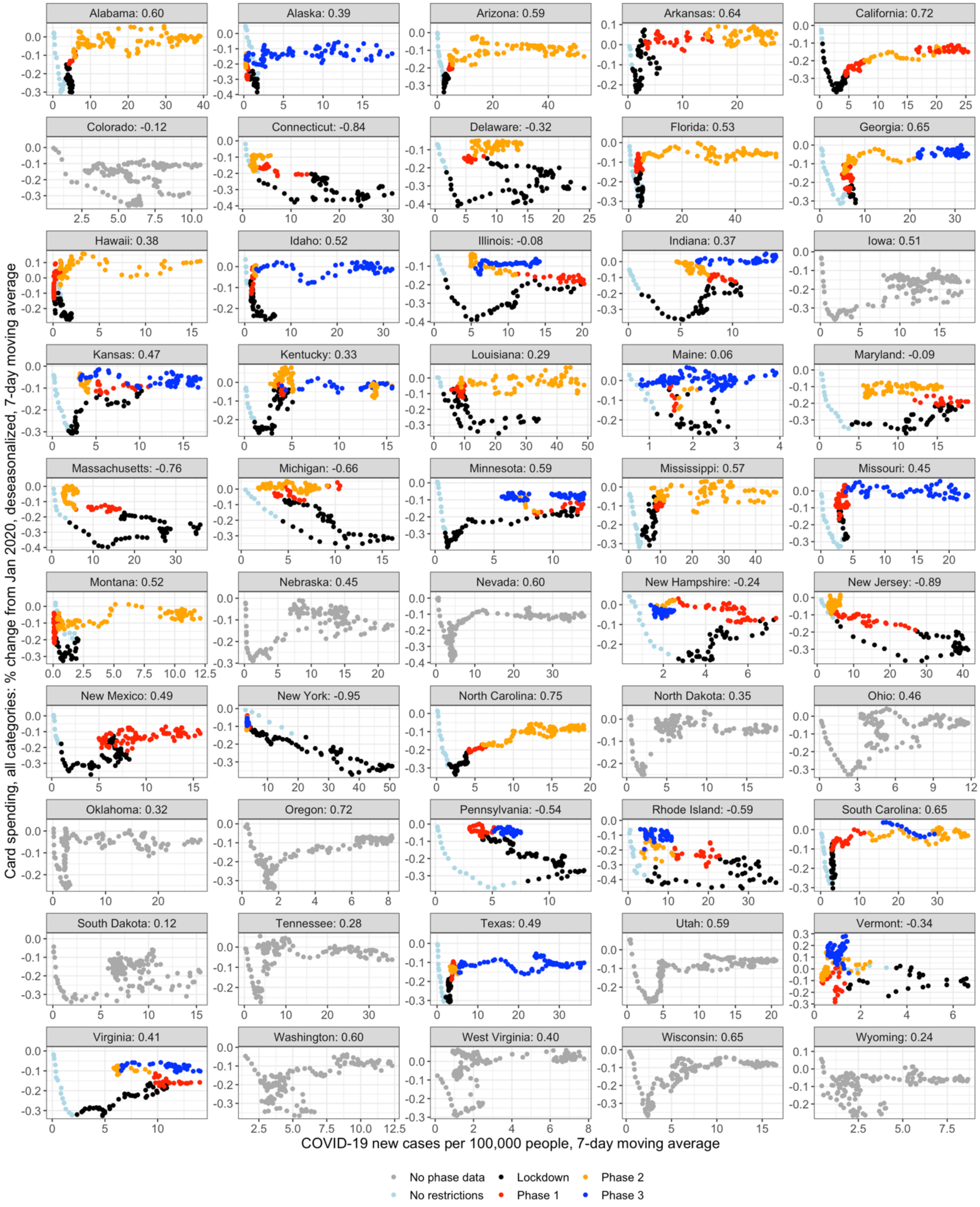

Figure S3. Seven-day moving average of de-seasonalized change in consumer card spending (relative to January 2020) as a function of seven-day moving average of new daily COVID-19 cases per 100,000 people.

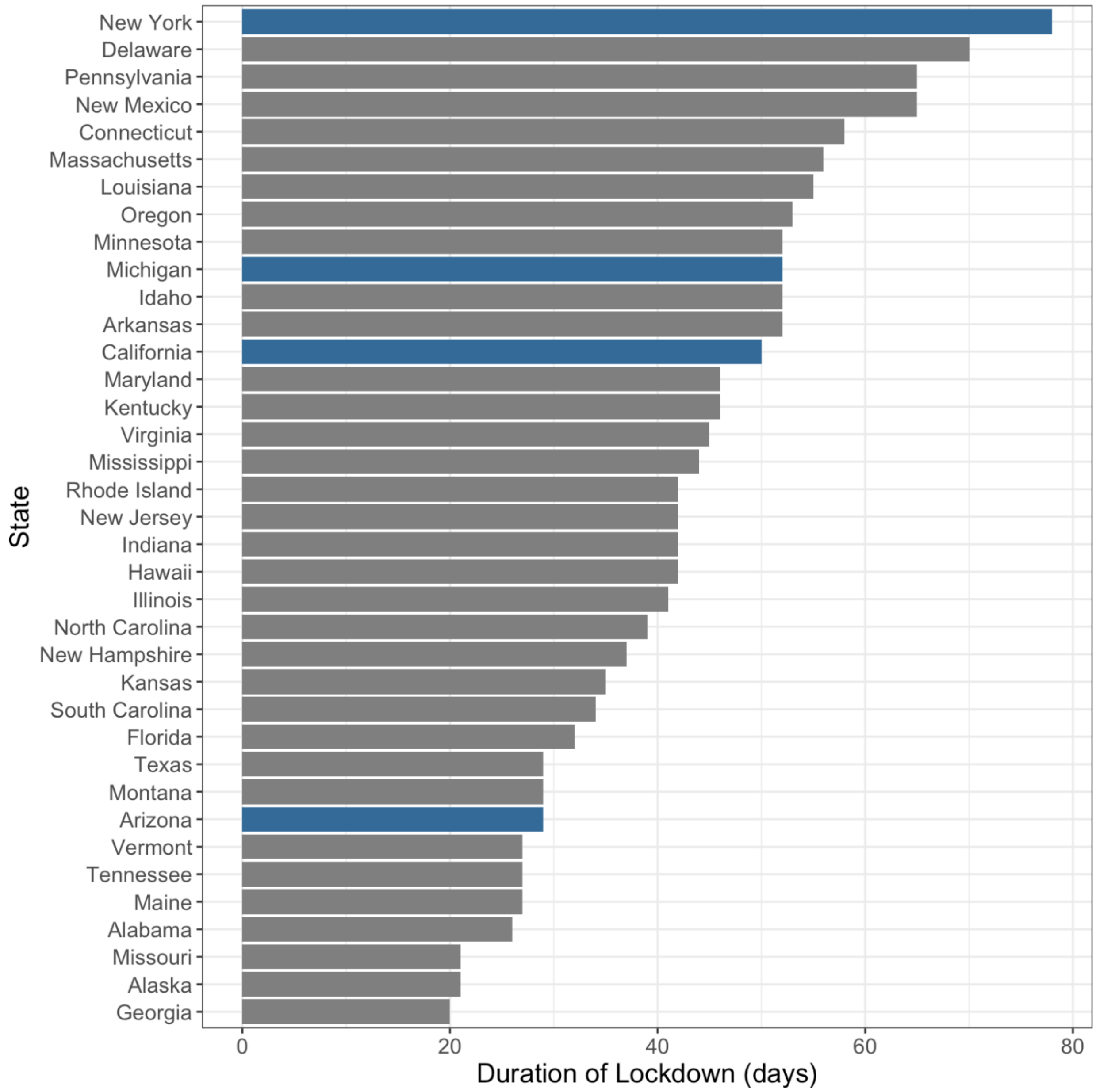

Figure S4. Duration of lockdown (in days) per state between March 13 and August 9, 2020 for the 37 states where we were able to identify state transitions. The states highlighted (blue) are the examples described in Fig. 1.

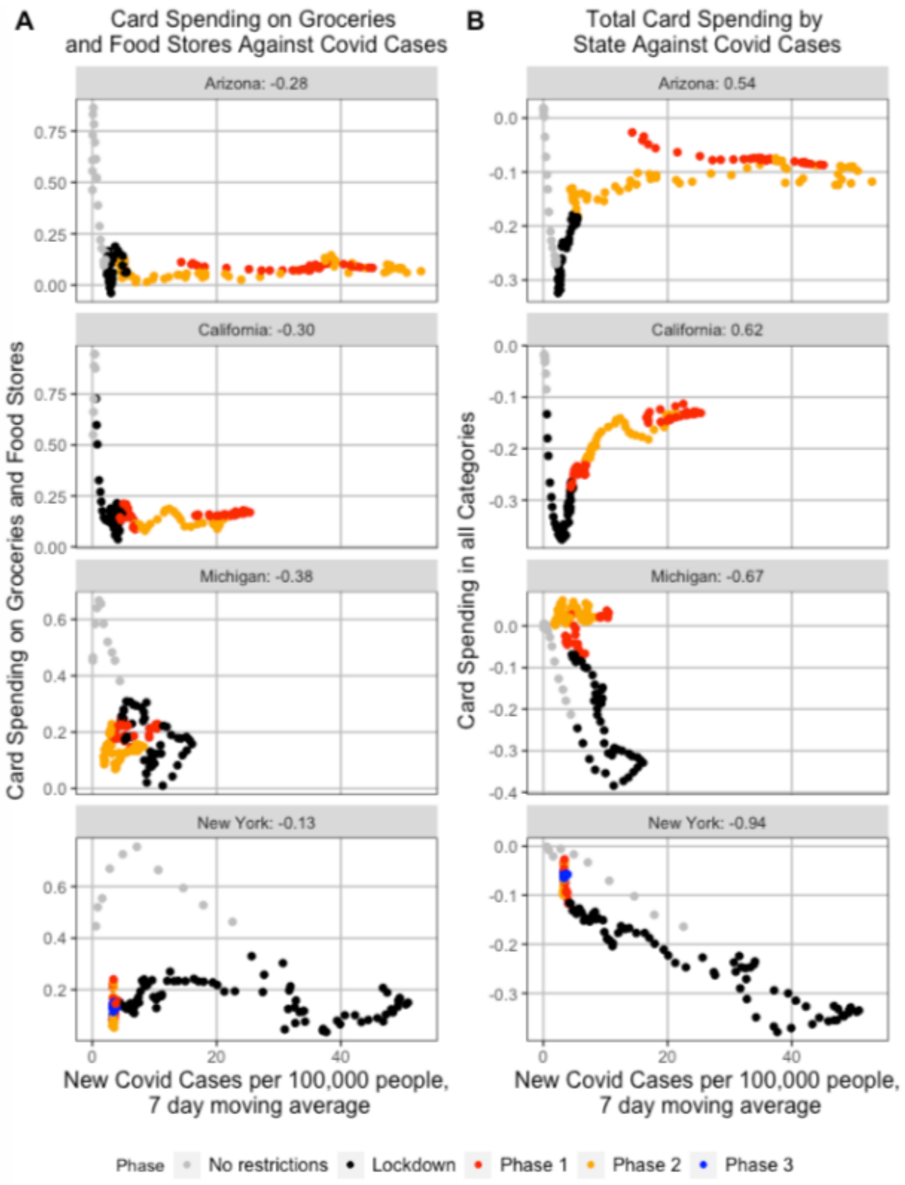

Figure S5. (A) Seven-day moving average of de-seasonalized change in consumer card spending on groceries and food stores (relative to January 2020) as a function of seven-day moving average of new confirmed daily COVID-19 cases per 100,000 people. (B) Seven-day moving average of de-seasonalized change in consumer card spending (all categories relative to January 2020) as a function of seven-day moving average of new confirmed daily COVID-19 cases per 100,000 people, in the period between March 13 and August 9, 2020 for New York, Michigan, California and Arizona. The colors represent our classification of state policies into lockdown and the three phases of the White House/CDC reopening guidelines.

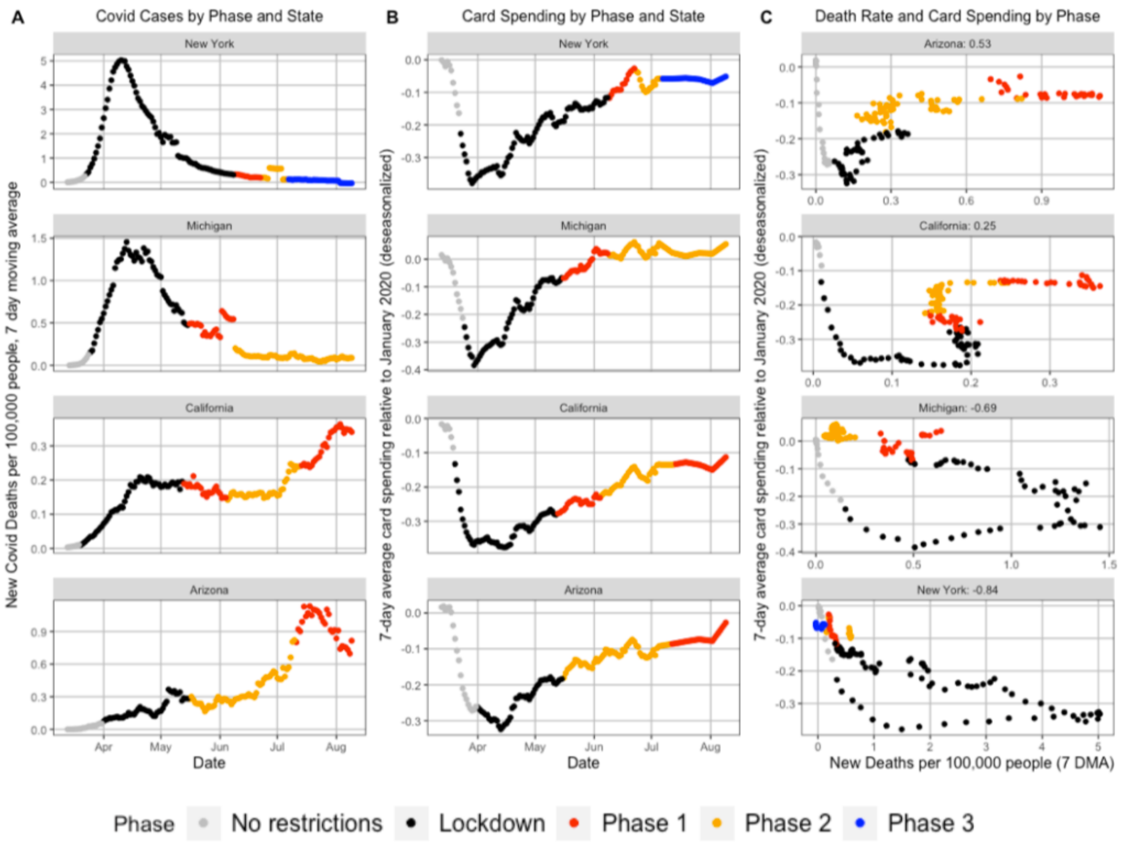

Figure S6. Temporal Patterns between March 13 and August 9, 2020 for New York, Michigan, California and Arizona of (A) seven-day moving average of daily COVID-19 deaths per 100,000 people over time. (B) seven-day moving average of de-seasonalized change in consumer card spending (relative to January 2020) over time. (C) Consumer card spending as a function of COVID-19 deaths. The colors represent our classification of state policies into lockdown and the three phases of the White House/CDC reopening guidelines. The Pearson correlations in (C) are -0.84 for New York, -0.69 for Michigan, 0.25 for California, and 0.53 for Arizona.

Fig. S7 shows the autocorrelation function of consumer card spending for the states that reopened in late April 2020. The leftmost bar in each chart shows that autocorrelation for 1-week lag is above 0.5 in most states, indicating that consumer card spending in a week is highly correlated with spending in the week before. The strong autocorrelation requires corrections in the DiD estimator such as averaging over time recommended in [2] and performed in this paper.

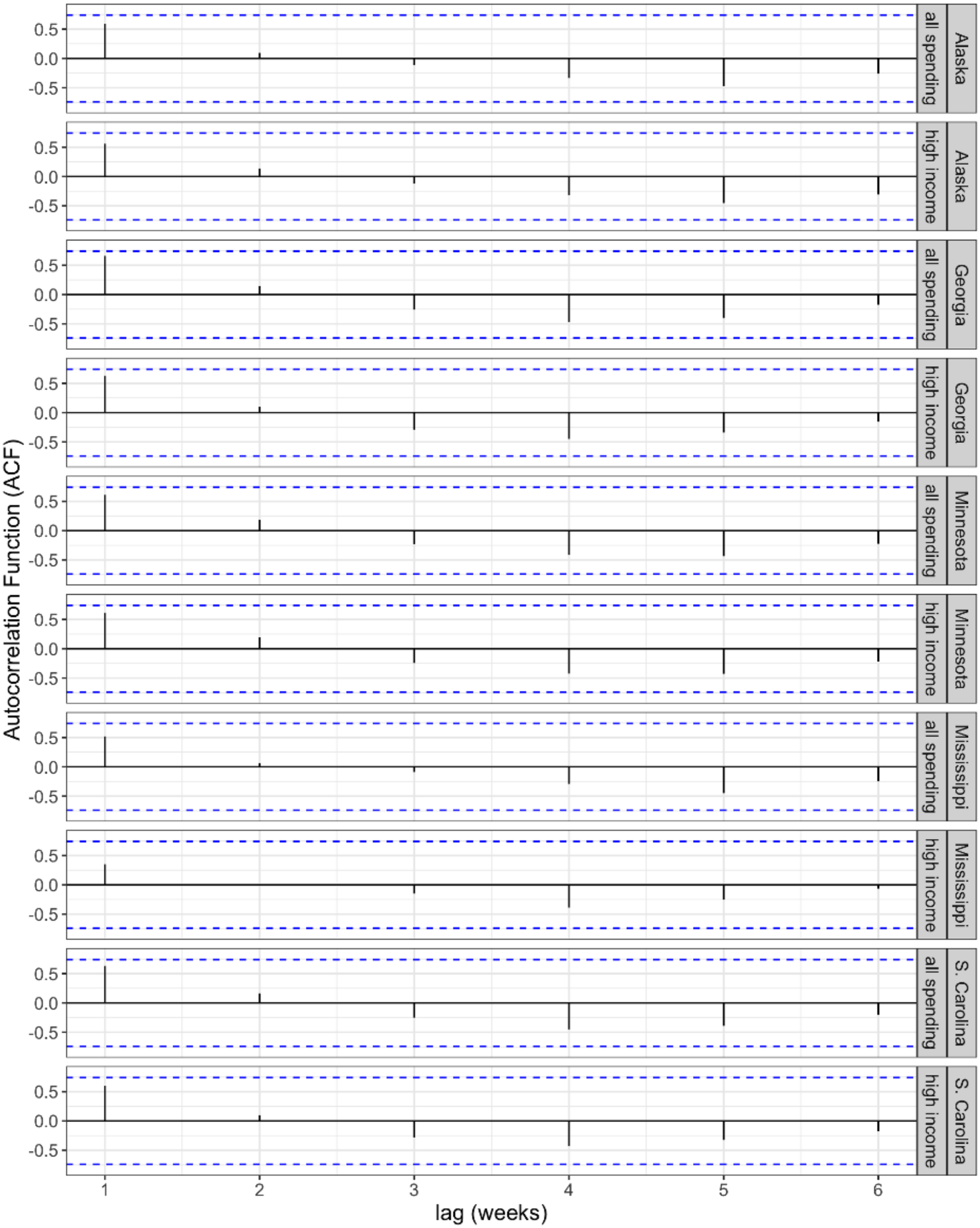

Figure S7. Autocorrelation in consumer card spending for the period three weeks before and three weeks after April 24th, 2020. The states shown are the "reopening" states considered in the difference-in-differences analysis. "All spending" refers to overall changes in consumer card spending (relative to January 2020). "High income" refers to the change in card spending by consumers living at zipcodes in the top quartile of median income.

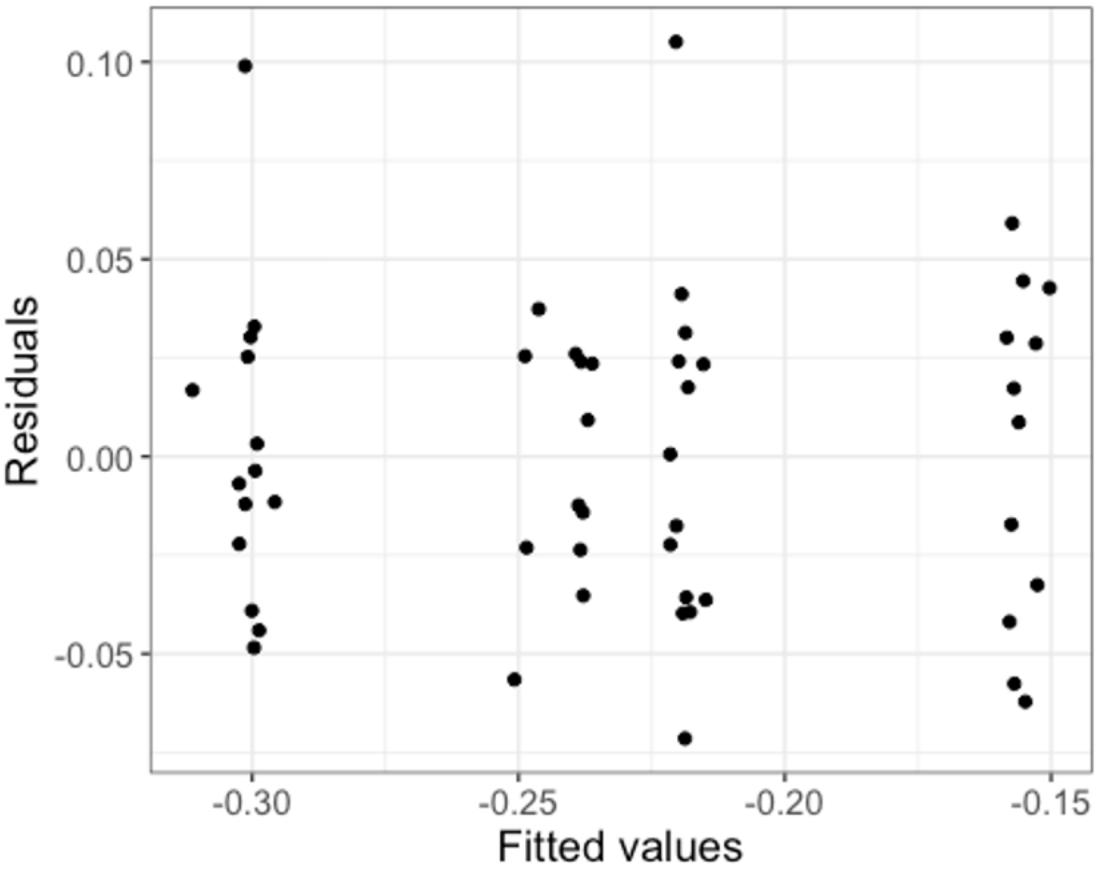

Figure S8. Scatterplot of the residuals of the difference-in-differences panel regression of overall consumer card spending – model (1) of Table I, as a function of the fitted values of the change in consumer card spending. The plot suggests that the variance of the residuals is approximately constant across fitted values. This suggests homoskedasticity of the residuals, and therefore the standard errors are not underestimated.

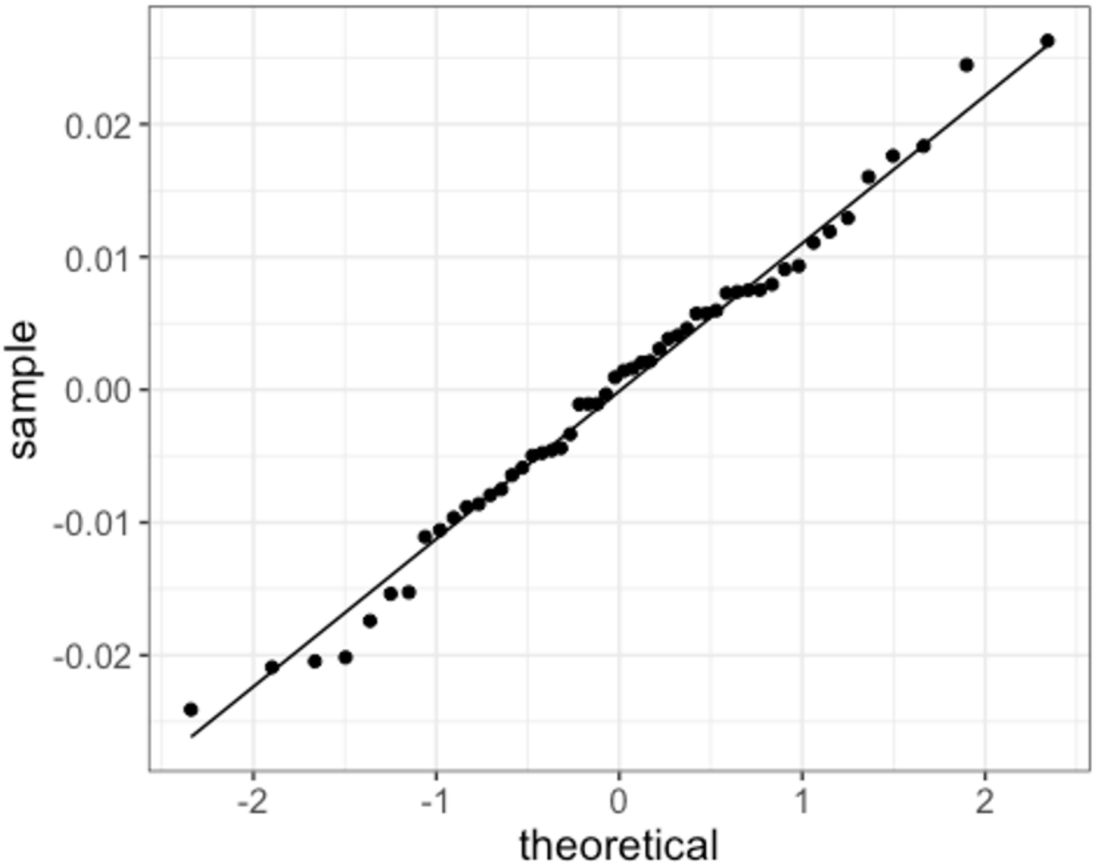

Figure S9. QQ plot of the residuals of the difference-in-differences panel regression of overall consumer card spending – model (1) of Table I. Since the regression residuals are approximately distributed on a straight line, the plot indicates that the residuals are normally distributed, and therefore the regression estimates (especially the standard errors) are reliable.

S.3. References

1. Brauner JM, Mindermann S, Sharma M, Johnston D, Salvatier J, Gavenčiak T, et al. Inferring the Effectiveness of Government Interventions Against COVID-19. Science (80-). 2020 [cited 25 Jan 2021]. doi:10.1126/science.abd9338
2. Bertrand M, Duflo E, Mullainathan S. How Much Should We Trust Differences-In-Differences Estimates? Q J Econ. 2004;119: 249–275. doi:10.1162/003355304772839588
