# Supplementary material for: Relationship among state reopening policies, health outcomes and economic recovery through first wave of the COVID-19 pandemic in the U.S
Source: PLoS One. 2021 Nov 18;16(11):e0260015. doi: 10.1371/journal.pone.0260015 (PMC8601438; doi:10.1371/journal.pone.0260015)
Supplement: Attachment — Submitted filename: Response to Reviewers.pdf [file pone.0260015.s002.pdf]

Authors’ Response to Editor and Reviewers

⇒ *We thank the Editor and Reviewers for the insightful comments. We have done our best to address all the suggestions and questions. In this document, our responses to each comment are preceded by the ⇒ symbol, and the consequent revision of the manuscript is marked with track changes.*

Editor Comments:

Regarding Reviewer #1 following comment:
Second, one of the big arguments is that it's not so much infections per se driving down consumption, but fear about transmission that depresses expectations and consumer confidence. What is your answer to that? One way is to compare states that are similar in political affiliation, but different in state policies. But, that goes back to #1 in needing controls and placebos.

I think that it is an important point to explore, but it will be more interesting to look at it from a different perspective than political affiliation.

⇒ *We thank the Editor for the suggestion. In the revised version we have performed a quantitative assessment of the effect of reopening policies conditional on political affiliation, median household income and other factors. Please refer to the response below to Reviewer #1 for more details.*

Journal Requirements:

When submitting your revision, we need you to address these additional requirements.

1. Please ensure that your manuscript meets PLOS ONE's style requirements, including those for file naming. The PLOS ONE style templates can be found at

https://journals.plos.org/plosone/s/file?id=wjVg/PLOSOne_formatting_sample_main_body.pdf and

https://journals.plos.org/plosone/s/file?id=ba62/PLOSOne_formatting_sample_title_authors_affiliations.pdf

⇒ *We have modified the formatting of the author list, headings and citations and other elements to meet PLOS ONE requirements.*

2. Please include captions for your Supporting Information files at the end of your manuscript, and update any in-text citations to match accordingly. Please see our Supporting Information guidelines for more information: http://journals.plos.org/plosone/s/supporting-information.

⇒ *We have added the SI captions and re-formatted citations to meet PLOS ONE requirements.*

Reviewer #1: Dear Authors:

Thank you for your work in this paper. I am in full agreement that state policies had much more to do with the fluctuations in consumption than the actual infection rate, but the execution of the statistical work in the paper needs an overhaul.

First, there is zero discussion of causal inference. If your goal is simply to say that consumption is better explained by state policies than by infections, then yes you can win that -- but that's already a fairly obvious point that we can see just by observing stories in the press. The trillion dollar question is how much the different policies have contributed to slower versus faster consumption growth, particularly in the length of time that the restrictions were imposed and their severity. But to do that, you at least need to control for confounding factors. Obviously there is only so much you can do -- there is too much time-varying heterogeneity -- but still some you can control for.

⇒ *We thank Reviewer 1 for all suggestions and questions. To address this, we keep the discussion that spending is more a result of reopening policies of states than it is a result of incidence rate in given areas.*

⇒ *In this revision, however, we complement that discussion with difference-in-differences (DiD) analyses to estimate the effect of the reopening in certain states on card spending, which is described in the Method Sections (3.5) and added to the Results section (4.4.1) of the manuscript. Our DiD regression analysis is based on 5 US states where Chetty et al. (2020) identified reopening policies in late April 2020. For these states, we computed how much card spending changed after state reopening. These changes were then compared with 21 states that did not reopen their economies during the same period. While all states experienced a sharp decrease in card spending during the lockdowns (around -30% in March relative to January 2020), we have found that states that did not reopen by late April reverted 8% of that decline, on average, between early April and mid May. On the other hand, card spending in the states that reopened recovered 1.5% more than the ones that did not reopen. This effect of reopening is statistically significant, and we also argue its magnitude is relevant, since it represents an elasticity of 1.5% / 8% = 19%.*

⇒ *While DiD does not capture all time-varying heterogeneity among states, it quantities the difference between states that reopened and states that did not*

reopen in the period above. Moreover, we have controlled for some of the fixed heterogeneity among states such as political majority and median income. We agree with the reviewer that not all confounding factors can be incorporated, and this is noted in the section about Limitations.

⇒ *In addition to overall card spending, we also run the DiD analysis considering card spending from the top income quartile of households for each state examined. For spending among higher income families, we have found an even larger effect. States that did not reopen by late April reverted 7.7% of the decline in card spending, on average, between early April and mid May, while spending in the states that reopened recovered 2.1% more than the ones that did not reopen, representing an elasticity of 2.1% / 7.7% = 28%.*

⇒ *Both of the DiD effects described above are significant at the 5% level. The manuscript describes the analyses above in detail.*

Second, one of the big arguments is that it's not so much infections per se driving down consumption, but fear about transmission that depresses expectations and consumer confidence. What is your answer to that? One way is to compare states that are similar in political affiliation, but different in state policies. But, that goes back to #1 in needing controls and placebos.

⇒ *In the previous version of the manuscript, we do discuss that fear is a result of government mandates in the form of NPI, which have a strong effect on consumer spending, which is consistent with Goolsbee and Syverson (2020). To complement that discussion, we have now included the political majority of each state into our DiD regression analysis as a control variable. Our regression results indicate that reopening have a causal effect on consumer spending trends, as described in the response to #1, but there is no significant effect of political majority.*

Third, the writing made it a little hard to read -- wordier in some places than it needed to be. There are also more citations you should make to recent work on consumption. Guerrieri et al have a paper on aggregate demand and supply from the pandemic; Yannelis and Pagela have a paper that came out at some point on consumption; etc.

I think you're on the right track, but it would be important to address these dimensions in your work going forward.

⇒ *We have simplified the writing, and made additional references to recent work on consumption such as Yannelis and Pagela et al., Guerrieri et al., del Rio-Chanona et al., and Eichenbaum et al., and more broadly to Bertrand et al. and others. In the manuscript we added that Guerrieri et al discussed widespread demand effects even though supply shocks were mostly concentrated in specific industries. We point out similar trends in consumer spending in the manuscript,*

though we make note of the importance of policy in consumers' decision to spend or not to spend. We also make note of the epidemiological models of consumer behavior discussed by del Rio-Chanona et al. and Eichenbaum et al. This is also consistent with our argument that NPI can be cause for reduced spending, as the fear factor associated with lockdowns being associated with people spending less.

Reviewer #2: Comments to the Author

The authors explored spatial temporal patterns of COVID-19 incidence and NPIs and their relationship with indicators of economic activity in the US. Their results suggest that consumer spending patterns can be attributed to government mandates rather than COVID-19 incidence in the states.

This is a comprehensive and ambitious research project, representing a potentially useful and novel contribution to the literature. The paper is well written and interesting. However, I have some concerns about the methods used. My main concerns are about the choice of the indicators.

1. The authors should provide further background and clarification on the choice of the incidence of COVID-19 as the main indicator of the spread of COVID-19. It is well known that States/Cities consider multiple other indicators to systematically monitor the status
eg https://www.cdc.gov/mmwr/volumes/69/wr/mm6934e3.htmhttps://forward.ny.gov/metrics-guide-reopening-new-york.
As a consequence, pros and cons versus other indicators considered by States/Cities to change NPIs policies (eg, test positivity rate, hospital admissions vs capacity, viral transmissibility) should be discussed within the limitations of this research, included in a sensitivity analysis and/or mentioned for readers awareness and future research.

- ⇒ *We thank Reviewer 2 for all suggestions and questions. We chose COVID-19 incidence as a metric because of its overall availability in publicly available datasets such as the New York Times and other sources. The choice of incidence is consistent with the CDC recommendation for states to track the trajectories of COVID-19 infections along with other metrics. However, for the analysis in this paper we considered that positivity rates don't necessarily account for the overall spread, since it depends on testing capacity.*
- ⇒ *Death-based metrics are arguably important, but in our analysis they would reveal similar patterns as those we discussed with COVID-19 incidence, as indicated in Fig. S6 of the Supplementary Information.*
- ⇒ *While there may be limitations in using only one metric to gauge the severity of COVID-19 in a given area, incidence was sufficient for our purposes of comparing trends across states.*

2. I would invite the authors to reflect on the factors that explain a higher incidence of COVID-19 (eg, socio-demographic composition, access to testing, public transportation, variants) and if / how those can affect the relationship with the rest of the indicators being analyzed at State level.

⇒ *In the new revision of the manuscript, we have included difference-in-differences (DiD) regression analyses to estimate the impact of reopening policies on spending. In these analyses, as control variables we have included socio-demographic and economic factors that explain part of the heterogeneity among states, such as income demographics and political majority. As per your suggestion, the inclusion of these factors enabled us to find that the effect of reopening policies on card spending is affected by household income but less by political majority. While the scope of this paper does not include determining the factors that drove the first wave of incidence, we thank the reviewer and are considering their suggestion as the subject for another paper.*

3. Once the background on the validity of COVID-19 incidence as an indicator of spread has been further elaborated, I suggest the authors should justify the choice of the source of data and clarify how the incidence of COVID-19 is defined:
• What is the relationship of the New York Times dataset to national and state surveillance efforts CDC eg Geographic Differences in COVID-19 Cases, Deaths, and Incidence — United States, February 12–April 7, 2020 | MMWR (cdc.gov)?
• Is the definition of COVID-19 incidence exclusively related to new, lab confirmed cases, excluding probable cases? Are asymptomatic cases included?
• Is it important for this exercise to consider geographical differences in case detection and reporting?

⇒ *As reported by both the Council of State and Territorial Epidemiologists (CSTE) and NYT, COVID-19 incidence includes confirmed cases and probable cases (https://cdn.ymaws.com/www.cste.org/resource/resmgr/ps/positionstatement2020/Interim-20-ID-01_COVID-19_NO.pdf). Confirmed cases include confirmed lab tests and probable cases include meeting clinical and epidemiological evidence without a test. The NYT definition of confirmed cases (https://github.com/nytimes/covid-19-data/blob/master/README.md) includes those tested positive in a lab and reported by a federal, state, or territorial government. Their definition of probable cases includes individuals who didn't have confirmed tests but were evaluated by health officials using guidelines created by states and the federal government health department. Antibody tests are considered evidence towards probable cases but are not sufficient on their own to count a probable case. It is unlikely that asymptomatic cases are included in these definitions unless a lab test result has been produced. These clarifications are added to the manuscript.*

4. I have similar comments as above for the choice and definition of card spending as a proxy for consumer spending. To further justify its validity as a measure of spending during the pandemic, the authors should clarify:
• What type of consumer expenses are pooled in that indicator (travel, transportation, food, entertainment, real estate, health, etc), in what composition.
• Why a pool of different types of expenses is the right proxy for the aims of this research; did the authors consider sensitivity analyses to a more restricted group - is the food analyses presented in Supplements restricted to essential purchases in supermarket, excluding restaurants and bars?
• Have the authors considered a sensitivity analyses focused on personal savings rates instead?

⇒ *Card spending reflects changes in aggregate card transactions processed by a major US operator and includes accommodation and food service; arts entertainment and recreation; general stores apparel and access, grocery/ food stores; healthcare and social assistance; transportation and warehousing, sporting goods and hobby, home improvement centers; and general merchandise. As indicated by Chetty et al (2020), this indicator of card spending is a reasonable proxy for consumer spending, and indirectly for economic activity during the pandemic. In the manuscript, this is described in section 3.2.*

⇒ *We thank Reviewer 2 for the question about a more restricted group. We have performed separate analysis for card spending overall and for a more restricted group – spending by households located in zip codes in the top quartile of income in each state. In the latter we found a significantly greater effect of reopening policy. Conversely, Chetty et al (2020) found that lower income households experienced a less elastic change in spending, which would make it harder to detect specific effects of reopening policy. (A similar rationale probably applies to essential categories such as supermarkets.)*

⇒ *The datasets used do not include personal savings rates, unfortunately. In the new revision, however, we have included a related factor – household income. While differences in median state income do not explain the effect of reopening policies on card spending, there are differences in such an effect between household income quartiles within states.*

5. With regards to the analyses of NPIs, I would advise the authors to also refer to the fact that there are multiple combinations of NPIs measures that are associated with different effectiveness. This element might have affected States differently, too, on top of overall NPIs stringency and compliance.

⇒ *Our classification of NPIs is a standardization effort based on information publicized by state authorities and news outlets, gathered from each state. This manually gathered information includes events such as restaurant restrictions, mask mandates, etc. which were heterogeneously publicized from state to state. This was because many states enacted different NPI at the city level and all the way down to the county level. Thus, our NPI classification dates include*

averaging across counties and cities within a state. This is described in the methods section of our paper in more detail in section 3.3 and in the Limitations section.

6. Potential suggestion to the authors: it may not be within the scope of this research, but I was curious if you conducted any exploratory quantitative threshold analyses across NPIs levels or phases eg what % change (range) in consumer spending was observed during the lockdown (vs pre-lockdown), and so on during phases of moderate and complete NPIs relaxations.

⇒ *In the new revision of the manuscript, we estimate the impacts of reopening on spending as a % change derived from the difference-in-differences (DiD) regressions, which quantifies how much of the change occurs in states that reopened as opposed to states that did not reopen in a given period. There were few instances where this type of test for causality were possible due to the short overlap in reopening status from state to state. This new analysis is included in section 3.4.1.*
