# Supplementary material for: Relationship among state reopening policies, health outcomes and economic recovery through first wave of the COVID-19 pandemic in the U.S
Source: PLoS One. 2021 Nov 18;16(11):e0260015. doi: 10.1371/journal.pone.0260015 (PMC8601438; doi:10.1371/journal.pone.0260015)
Supplement: Attachment — Submitted filename: Response to Reviewers.pdf [file pone.0260015.s003.pdf]

Authors' Response to Editor and Reviewers

⇒ *We thank the Editor and Reviewers for the comments about this second review. We have done our best to address the suggestions and questions. In this document, our responses to each comment are preceded by the ⇒ symbol, and the consequent revision of the manuscript is marked with track changes.*

Editor Comments:
Mainly address the first comment of reviewer 1.

⇒ *We thank the Editor for handling the submissions and reviews. Our responses to Reviewer 1 are listed below.*

Reviewer #1: Thank you authors. I think this is a good improvement. I am on board with the econometric approach and results, but want to flag two important areas for this to be more presentable.

First, substantively: are you controlling for time and state fixed effects in your DD regression? I could not tell by the table formulation and your discussion of them. This is important. Put the temporal fixed effects in at least with the month and year aggregation. You need to isolate variation within the same state over time. To further drive home your point, you could always do a mediation analysis (R has a nice package) where you look at how the unemployment rate may serve as a mediating factor. The main point here is to ensure you're doing the DD right since those coefficients look a little big.

⇒ *We thank Reviewer 1 for all questions, comments and suggestions. We are controlling for time and state fixed effects in the DD regression. This is better described on the notes below the results table. The temporal fixed effects are aggregated at the week level. This is because we are looking at the short-term effect of the first wave of reopenings – 2 to 5 weeks before and after late April 2020. We did not include data before April because that would include the shock of the nationwide lockdown in late March. Likewise, we didn't include more than 5 weeks after late April because that would include the summer, which was plagued by a second wave in some states. Moreover, this choice of time windows enables us to compare its similarity with Chetty et al., who found a somewhat similar DD coefficient for a similar specification. (Note, however, that our discussion of the implications differs from theirs.) Moreover, we did control for state fixed effects. In lieu of a mediation analysis of causal mechanisms, we opted for a (perhaps simpler) analysis of alternative specifications of the causal inference of the effect of state reopenings on spending. These altenatives included adding political affiliation, income and population density as controls,*

varying the number of weeks included in the regression before and after the late April reopening, varying the set of control states, and aggregate observations at the county vs. state levels. In most models, the DD coefficient indeed resulted smaller than the ones reported in the previous revision, especially when all 50 states are included in the regression.

Second, presentation: the plots and tables don't look as good as they could. I know it might sound trite, but just take a look at tables in top economics journals, like QJE/AER. You want to make clear the coefficients of interest, have table notes that describe what you're doing (and the controls that are included), the sample period, etc. The tables you have for the DD are not at all conventional -- basically the best thing to do is an event study where you're plotting the coefficients on the post period 1 month after the removal of a lockdown all the way up to however many months you care about (maybe 6), with some before months too. You also want to put confidence intervals on them and ensure you're clustering at the state level.

⇒ *We have reformatted the tables (in a format similar to QJE) to list the coefficients in the relevant order, and to include the results of interest including significance levels, standard errors, number of observations, CI, etc. Moreover, the table notes were expanded to clarify, for each model in the table, the fixed effects, period included, treatment and control states, additional control variables, level of aggregation, etc.*

⇒ *Standard errors are clustered at the state level. This is also described in the table notes.*

(note that if you're putting in state FE etc, make sure you have all 50 states -- clustering with less than 40 obs [and here it's the states that matter] will give biased estimates)

⇒ *We have all 50 states in all models, except the models that replicate the specification described in Chetty et al (5 treatment + 21 control states). As noted above, considering all 50 states result in DD coefficients that are smaller than the ones reported in the previous revision.*

Reviewer #2: I appreciate the comprehensive responses provided to all of my questions and comments. The resulting changes in the manuscript are clear and exhaustive. The paper appears in very good shape now and I recommend its acceptance for publication in PLOS ONE.

⇒ *We thank Reviewer 2 for the questions, comments and suggestions included in the first review, and the feedback provided in the current one.*

Journal Requirements:

Please review your reference list to ensure that it is complete and correct. If you have cited papers that have been retracted, please include the rationale for doing so in the manuscript text, or remove these references and replace them with relevant current references. Any changes to the reference list should be mentioned in the rebuttal letter that accompanies your revised manuscript. If you need to cite a retracted article, indicate the article's retracted status in the References list and also include a citation and full reference for the retraction notice.

⇒ *The following references were added to the previous revision for better characterization of the analysis of causal inference:*

MIT Election Data and Science Lab. County Presidential Election Returns 2000-2020. Harvard Dataverse; 2020. doi:10.7910/DVN/VOQCHQ

USDA Economic Research Service. Unemployment and median household income for the U.S., States, and counties, 2000-20. 2021 [Accessed 4 Oct 2021]. Available: https://www.ers.usda.gov/data-products/county-level-data-sets/download-data/

Callaghan T, Lueck JA, Trujillo KL, Ferdinand AO. Rural and Urban Differences in COVID-19 Prevention Behaviors. J Rural Heal. 2021;37: 287–295. doi:10.1111/JRH.12556
